# Longitudinal relationships between school assets, traditional bullying, and internet gaming disorder: the role of self-control and intentional self-regulation among Chinese adolescents

**DOI:** 10.3389/fpubh.2023.1162022

**Published:** 2023-07-10

**Authors:** Ke-Nan Qin, Xiong Gan

**Affiliations:** Department of Psychology, College of Education and Sports Sciences, Yangtze University, Jingzhou, China

**Keywords:** school assets, intentional self-regulation, self-control, traditional bullying, internet gaming disorder, Chinese adolescents

## Abstract

**Introduction:**

Although developmental assets have been proven to be enabling factors for both adolescent traditional bullying and internet gaming disorder (IGD), there is a lack of empirical evidence that has investigated the direct relationship between school assets and both of these problematic behaviors concurrently. Based on the positive youth development (PYD) perspective, the present study aimed to explore the relationship between school assets, intentional self-regulation (ISR), self-control, traditional bullying, and IGD among Chinese adolescents.

**Methods:**

A total of 742 middle school students (M_age_ = 13.88 years, SD = 1.99 years) were followed up to measure school assets, ISR, self-control, traditional bullying, and IGD in two waves that were separated by 5 months.

**Results:**

Structural equation modeling (SEM) indicated that T1 school assets negatively predicted T2 traditional bullying and T2 IGD. T1 self-control significantly mediated the relationships between T1 school assets and T2 traditional bullying, as well as between T1 school assets and T2 IGD. Additionally, T1 ISR strengthened the positive effect of T1 school assets on T1 self-control and further moderated the two mediating paths.

**Discussion:**

These findings show that plentiful school assets support the development of self-control and are more successful in reducing traditional bullying and IGD, particularly among students with higher ISR. As a result, schools should take measures to provide superior-quality assets for the positive development of youth, which will help to prevent and relieve traditional bullying and IGD in the school context.

## Introduction

Traditional bullying and internet gaming disorder have become severe public health problems, especially among the adolescent population. A study used the data of 3,675 Chinese children and adolescents from a national representative survey and reported the prevalence of traditional bullying at 17.3% (1). Han et al. (2) investigated 3,777 students from 28 schools in China and found that those who reported being bullied, bullying others, or witnessing bullying were 26.10, 9.03, and 28.90%, respectively. Traditional bullying refers to a particular type of aggressive behavior that has three characteristics: intentional injury, repetition of the behavior, and an imbalance of strength between both parties (3). Prolonged exposure to traditional bullying is harmful to the physical and mental health of individuals, whether they are bullies, victims, bully-victims, or bystanders (4). Specifically, in terms of psychological problems, teenagers engaging in bullying tend to be more likely to suffer from depression, anxiety (5), personality disorders (6), lower self-esteem (7), and life satisfaction (8), and more likely to generate suicidal ideation (9). They also exhibit a wider range of externalizing problem behaviors, including substance abuse, binge drinking, self-harm, suicide, and delinquency (10–13). Besides, Liao et al. (14) conducted a cross-sectional study design based on 6,379 adolescent game players from 34 provincial administrative districts in China and found that the prevalence of IGD was as high as 17%, which was much higher than many other countries (15). Internet gaming disorder (IGD) refers to an individual's uncontrollable, excessive, and compulsive use of online games that causes social and/or emotional problems (16). The WHO included it in the International Classification of Diseases (ICD-11) (17). Lee et al. (18) have demonstrated that IGD causes structural changes (e.g., aberrant posterior superior temporal sulcus functional connectivity) and functional impairments (e.g., executive dysfunction) in the adolescent brain. A comprehensive review has also shown that IGD is associated with psychopathologies such as depression, anxiety, social phobia, obsessive-compulsive symptoms, and attention deficit hyperactivity disorder (19). Meanwhile, teenagers who are addicted to IGD report higher levels of loneliness and hostility, lower self-esteem and wellbeing, and poorer social skills and academic performance (20–22). This suggests that involvement in traditional bullying and internet games has extremely serious impacts on adolescent development (4, 23).

Given that adolescence is a critical period of development and the high prevalence and negative consequences of traditional bullying and IGD during this time, researchers are committed to studying the characteristics and influencing factors that affect its occurrence. For example, through a longitudinal survey of 2,844 adolescents aged 11–15 years, Cho et al. (24) found that their bullying decreased over time, generally peaking in middle school and declining throughout high school. Another follow-up design based on a large sample of Chinese adolescents reported that adolescents' IGD tended to increase over time (25). Furthermore, some research has revealed that violence exposure and deviant peer affiliation have predictive roles in traditional bullying (26, 27). An empirical study has investigated many predictors of IGD, such as impulsivity and fear of missing out (28). Scholars stated that a lot of research on the outcomes of youth development paid more attention to teenagers' “what problems occurred” than their “what capabilities developed” (29), but in fact, this view was a bit excessively negative. Therefore, we will concentrate on exploring the positive antecedent variables of traditional bullying and IGD. From the positive youth development (PYD) perspective, this study will discuss the relationships between school assets and problematical behaviors, including traditional bullying and IGD in the Chinese context, which have both theoretical and practical significance for promoting adolescents' positive development and preventing and alleviating traditional bullying and IGD.

### School assets, traditional bullying, and internet gaming disorder

For adolescents, school has gradually replaced home as the prime place for their daily studies and lives. The PYD perspective states that adolescents are relatively plastic, and when external settings such as family, school, and community supply appropriate developmental assets, their developmental potential will be stimulated and favorably developed, which also applies to adolescents experiencing developmental problems (30). Chai and Lin (31) have suggested that school transitions may be an important opportunity for adolescents to achieve adaptive development. This shows that it is indispensable for us to investigate the impact of relevant assets in school situations on the youth's positive development. School assets, as proposed by Benson et al. (32), represent the state of adolescents' assets within the context of the school under the developmental assets framework, including many protective factors such as school climate, teacher-student relationship, school safety and norms, and school engagement. A recent longitudinal study has revealed that school assets of high quality can effectively improve teenagers' subsequent wellbeing (33). The stacking effect assumption of the developmental assets framework also points out that the more assets, the more beneficial to the individual (34). In other words, the more assets teenagers have, the less likely they are to have problematic and risky behaviors, which is conducive to the healthy development of the present and the future (35, 36).

Traditional bullying often occurs in the school context (37). A study showed that the prevalence of traditional bullying reported by students varies from school to school (2). Adolescence is also the peak period for the development of aggressive behavior. Researchers from various cultural backgrounds investigated the impact of positive factors in school contexts on adolescent traditional bullying and achieved many findings. For example, young people in a positive school climate reported less bullying (38). Han et al. (2) have also indicated that the indicators of school climate are protective factors for bullying in China, including individual perceptions of their teachers, peers, and academic performance. And both school satisfaction and school bonding significantly buffer the occurrence of bullying during adolescence (39). Wang et al. (40) found that teacher-student relationships effectively mitigated physical and verbal/relational bullying among middle school students. Conversely, a meta-analysis has revealed that there is a significant positive effect of conflictual teacher-student relationships on bullying perpetration (41). Moreover, Gan et al. (42), through a longitudinal survey, found that school resources predicted a decrease in subsequent bullying among Chinese youth. That is, school assets may be a negative predictor of traditional bullying.

Similarly, it has been noted that a high school climate plays a role in buffering problematic internet game use among Chinese adolescents (43, 44). Several prior studies have also shown that the positive factors in school contexts all contribute to protecting adolescents from the negative effects of IGD, such as teacher autonomy support, school engagement, and school connectedness (45–47). Additionally, some empirical evidence has suggested that developmental assets, including school assets, facilitate positive development and alleviate non-adaptive development among cross-cultural adolescents (32, 48). A number of studies have indicated that adolescents who have more assets tend to be less addicted to online games (42, 49). For instance, some researchers conducted a two-wave design in a sample of 1,023 adolescents and revealed that the developmental assets negatively predicted their IGD both concurrently and longitudinally in China (50). These suggest that good school assets could attenuate the likelihood of youth indulging in IGD. That is, school assets may have a negative predictive effect on adolescent IGD. To summarize, the present study hypothesizes that school assets negatively predict traditional bullying and IGD among adolescents (Hypothesis 1).

### The mediating role of self-control

Apart from the direct impacts of school assets on traditional bullying and IGD, the cognitive processes underlying the relationships between school assets and both remain unclear. The strength model of self-control implies that self-control may be an indirect mechanism between school assets and these two risky behaviors. This theory, proposed by Baumeister et al. (51), indicates that self-control depends on limited resources, which will be depleted by individuals in their acts of self-control. When resources are depleted to a certain level, further self-control is not possible, and the individual fails to control himself or herself. In other words, individuals tend not to choose to engage in traditional bullying and indulge in internet gaming when they have sufficient school assets to spend on maintaining their own consumption of self-control. Self-control is defined as the ability to override or change one's inner responses, as well as to interrupt undesired behavioral tendencies and refrain from acting on them (52). Several studies have demonstrated that failure of self-control leads to many personal and social problems, such as addiction, eating disorders and binges, emotional problems, underachievement in school and work, academic procrastination, violent behavior, and even criminal behavior (53, 54).

Extensive research evidence has shown that a favorable school environment or high-quality school resources are closely connected to high levels of personal self-control. For instance, adolescents may benefit from positive resources in the school environment to help them strengthen their own regulation (42). And young people in a protective school climate tended to develop the ability to control themselves better (55). Specifically, the more support from teachers and peers that teenagers have, the better they tend to exercise self-control as well (56). Correspondingly, deviant peer associations would present the opposite relationship with self-control in youth (57, 58). At the same time, Xiang et al. (49) conducted a longitudinal study and revealed that developmental assets positively predict subsequent adolescent self-control. Given the inclusion of school assets in the framework of development assets, school assets may contribute to the restoration of self-control. So, it is reasonable to assume school assets could predict increased self-control among adolescents.

According to the general theory of crime (59), self-control is a determinant of individuals demonstrating high levels of impulsivity and engaging in problematic behaviors, such as traditional bullying and IGD. Specifically, those with low self-control may not be able to appropriately manage their impulses and thus produce undesirable outcomes (60), while those with ideal self-control can timely supervise and amend themselves, are better at problem solving, and are more considerate of future consequences, thus effectively preventing the recurrence of problem behaviors (61). On the one hand, low self-control has long been identified as a crucial potential risk factor for bullying (62). A large body of empirical research has revealed that self-control is closely related to externalizing problems such as intrusion, violence, and criminal behavior and mitigates and reduces individual aggressive behavior (63, 64). Many researchers have also found that low self-control causes teenagers to perpetrate bullying against peers (24, 65, 66), and conversely, high self-control predicts a decrease in their traditional bullying (67, 68). Thus, self-control is not only related to traditional bullying but also negatively predicts traditional bullying. On the other hand, impaired control is also a primary cause of IGD and other addictive behaviors (69). Ample empirical evidence has indicated that self-control could lead to a reduction in the risk of internet addiction, smartphone addiction, and social media addiction (70–72). A meta-analysis designed to examine risk and protective factors for IGD has also found that self-control is the only protective factor strongly linked to IGD among Chinese people (73). Online gaming may make those with poor self-control more prone to compulsive and overuse behaviors, further leading them to indulge in IGD (74). Higher levels of self-control predicted fewer IGD symptoms in young people, according to findings from both cross-sectional and longitudinal research (49, 75, 76). Overall, self-control is associated with the development of IGD and also alleviates its symptoms.

In addition to the above theories and previous evidence for the separate paths (i.e., from school assets to self-control and from self-control to traditional bullying and IGD), some studies have demonstrated the mediating effect of self-control between high-quality assets in school and adolescent problem outcomes. Based on a sample of Chinese youth, Xiang et al. (49) showed the mediating role of self-control in the association between developmental assets and IGD. Among left-behind children and adolescents in China, self-control was found to mediate the relationships between positive school climate (e.g., teacher and peer support) and both their internalizing and externalizing problems (55, 56). Inspired by the above evidence, we speculate that school assets may protect youth against participating in traditional bullying by enhancing their self-control. In the same way, school assets may predict higher self-control among adolescents, eventually decreasing the risk of playing online games. In general, the present study hypothesizes that school assets indirectly affect traditional bullying through self-control (Hypothesis 2), and self-control also mediates the relationship between school assets and IGD (Hypothesis 3).

### The moderating role of intentional self-regulation

As aforementioned, school assets may contribute to a decrease in traditional bullying and IGD through self-control. However, the mediation effect may also vary from person to person. Intentional self-regulation (ISR) is a major form of self-regulation in adolescence and refers to a series of contextualized actions that are actively aimed toward harmonizing demands and resources in the context with personal goals to attain better functioning and enhance self-development (77). The developmental systems model emphasizes that development is a matter of the relationship between the individual and the environment and that individuals actively engage with and make healthy and supportive connections with the context (78). That is to say that individual behavior is a result of the positive two-way interaction between them and their environment, namely that their own factors (ISR) interact with environmental factors (school assets) to jointly influence their development (79, 80). This implies the theoretical feasibility of examining the moderating effect of ISR. ISR is an internal asset because it reflects the individual's subjective initiative. On the one hand, a higher ISR could buffer the negative events experienced by individuals and prevent them from internalizing and externalizing problem behaviors, such as suicidal ideation, smoking behavior, and social network site addiction (81–83). On the other hand, it also strengthened the indicators conducive to positive development, including competence, confidence, character, connection, and caring (84, 85). Given that prior studies have more often examined the moderating role of ISR on negative behavioral consequences, this study aims to explore whether ISR is a moderator in the relationship between school assets and positive development outcomes (i.e., self-control) among adolescents.

**Figure 1:**
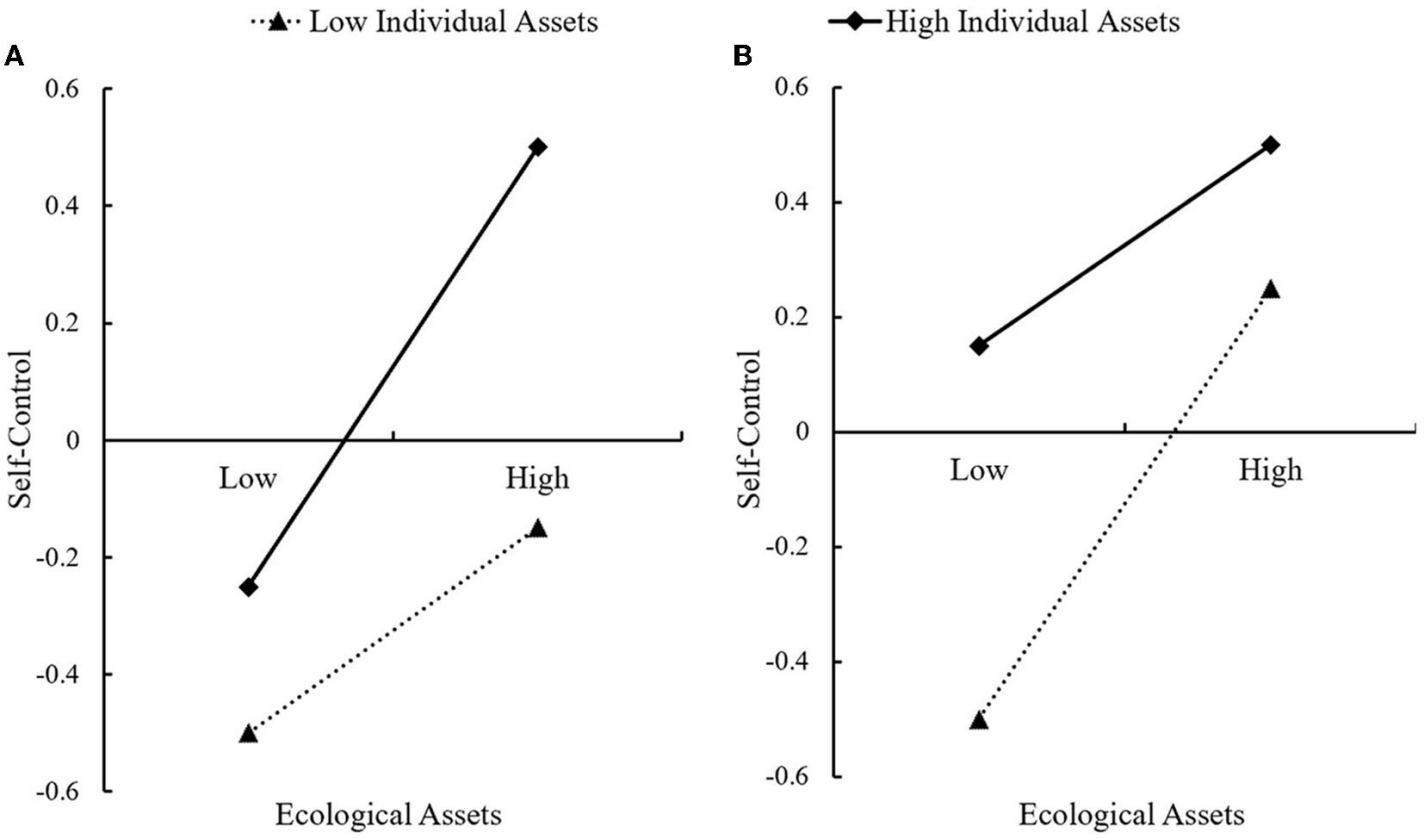
**(A, B)** Two patterns of interaction effects.

It is worth noting that there are two different patterns of individual characteristics (i.e., ISR) (86). The protective-enhancing hypothesis holds that one protective factor facilitates the effect of another protective factor on the outcome variables (87, 88). According to this view, the positive effect of school assets would be stronger with a higher ISR (see Figure 1A). In contrast, the protective-attenuating hypothesis maintains that one protective factor does not amplify but rather “undermines” the positive effect of another protective factor (86, 88). From this perspective, the beneficial effects of school assets are stronger with a lower ISR. Because when individuals are at high levels of ISR, self-control is almost always great (a ceiling effect occurs), and the advantageous effects of school assets are less likely to be evident (see Figure 1B). The two hypotheses above each imply different practical implications: the former indicates that the provision of abundant school assets would particularly benefit adolescents with higher ISR scores, while the latter shows that abundant school assets would only benefit those students with lower ISR scores. Hence, we aim to examine the pattern of interaction between school assets and ISR. The present study hypothesizes that ISR moderates the effect of school assets on self-control (Hypothesis 4) and further moderates the mediating effect of self-control between school assets and both traditional bullying and IGD (Hypothesis 5). For the two moderating patterns mentioned above, no explicit assumptions are made.

### The present study

Most previous studies used cross-sectional designs to examine the negative antecedent variables (e.g., violence exposure and impulsivity) of traditional bullying and IGD and their mechanisms of occurrence, and more often used manifest variable modeling to construct theoretical models (26, 28). Considering that, in terms of research content, we explore the protective effect of school assets on adolescents' problem behaviors and the buffering role of self-control and intentional self-regulation based on a positive psychology perspective. In terms of research methodology, this study proposes to use a latent variable model, which takes into account the effects of measurement error and better explains the relationships among multiple variables than traditional path analysis.

**Figure 2:**
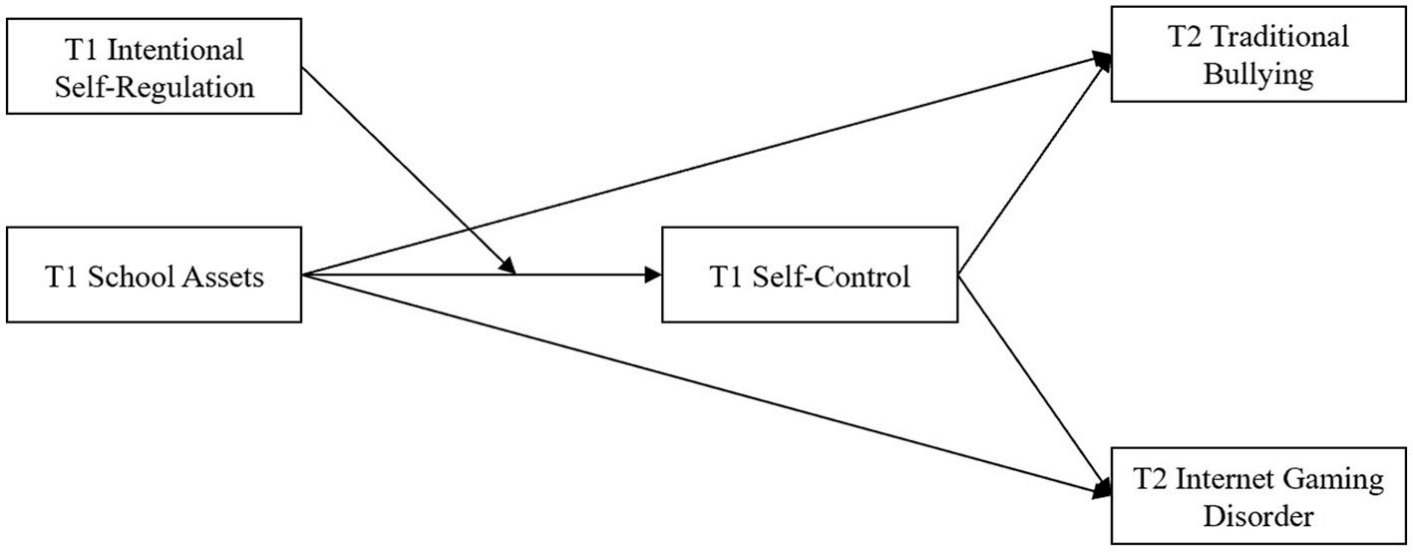
The hypothesized moderated mediation model.

To the best of our knowledge, there is a lack of evidence about the associations between school assets and both traditional bullying and IGD and the potential mediating and moderating mechanisms underlying the relationships. To address the research gaps and given the empirical studies and theories mentioned above, this study attempts to access the impact of early asset profiles (i.e., external assets: school assets; internal assets: ISR and self-control) on subsequent problem behaviors (i.e., traditional bullying and IGD) in Chinese youth. As demonstrated in Figure 2, the present study explores the relationship between school assets and adolescent problem behaviors and constructs a moderated mediation model through a two-wave design with the following hypotheses:

H1: school assets negatively predict adolescent traditional bullying and IGD;
H2: self-control mediates the association between school assets and traditional bullying;
H3: self-control mediates the association between school assets and IGD;
H4: ISR moderates the association between school assets and self-control;
H5: ISR moderates the mediating effect of self-control on the associations between school assets and traditional bullying, as well as between school assets and IGD.

## Method

### Participants and procedure

In this study, students were recruited to participate by employing random cluster sampling from three public middle schools in Hubei Province on mainland China. The first occasion of data collection took place at the beginning of the school year of 2021/22. All of the students were invited to respond to a questionnaire measuring adolescent school assets, ISR, and self-control using the paper-and-pencil method. Five months later, the students finished the questionnaires about development and problem behavior once again. At Time 1, a total of 822 students had participated in our study. The students ranged in age from 12 to 18 years and included 449 boys (54.62%) and 373 girls (45.38%). For various reasons, such as asking for leave and transferring, 80 participants dropped out. Finally, 742 students completed the survey at Time 2, resulting in an overall attrition rate of 9.73%. Among the matched sample (*N* = 742), with a mean age of 13.88 years (SD = 1.99), 395 (53.23%) were boys and 347 (46.77%) were girls. All procedures were approved by the Ethics Committee for Scientific Research at the corresponding author's institution before the survey was carried out. Likewise, the present study also received informed consent from school leaders and students. Trained teachers and research assistants explained to participants the important principles relevant to this study, including confidentiality, independence, voluntary participation, do-no-harm, and free withdrawal. Each time, the participants completed the questionnaire in the classroom during school hours in under 15 min. Researchers thanked the students and teachers for their cooperation after the data was collected.

## Measures

### School assets at Time 1

The school assets subscale of the Developmental Assets Profile (DAP) developed by the Search Institute (89) was employed to examine the perceived school assets of adolescents, such as engagement, safety, norms, and care in the school context. The scale contains ten items (e.g., “I have teachers who urge me to develop and achieve” and “I am trying to learn new things”). Responses to these items were rated on a 4-point Likert scale, ranging from 1 (not at all or rarely) to 4 (extremely or almost always), with higher average scores indicating richer school assets for youth. Scales (90) showed that DAP exhibited cross-cultural applicability. In previous studies, the school assets scale also showed good reliability and validity among Chinese adolescents (33). In this study, the Cronbach's alpha for the school assets subscale was 0.87 at Time 1.

### Intentional self-regulation at Time 1

The Adolescent Intentional Self-Regulation Questionnaire was used to assess the level of ISR among adolescents (91, 92). This questionnaire comprises nine items, which are divided into three dimensions: goal selection (e.g., “As long as I set a goal, I will stick to it to the end”), goal optimization (e.g., “In order to achieve the goal, I will try all kinds of ways and methods as much as possible”), and goal compensation (e.g., “When there are difficulties in the realization of the goal or the implementation of the plan, I will see what others do”). All of the participants were invited to respond to these items on a 5-point Likert scale from 1 (strongly disagree) to 5 (strongly agree), with higher average scores reflecting higher levels of ISR. The questionnaire has been proven to have good reliability and validity (33, 93). In our study, the Cronbach's alpha for this questionnaire was 0.80 at Time 1.

### Self-control at Time 1

We used the Brief Self-Control Scale (BSCS) to measure the level of self-control in youth (52). This scale consists of thirteen items (e.g., “I often take action without thinking carefully in advance.” and “I'm a little difficult to concentrate on”). All of the items were completed on a 5-point Likert scale, ranging from 1 (not like me at all) to 5 (very much like me). Among all of them, nine items are reverse-scored. Higher average scores represent higher levels of self-control in adolescents. In a prior study, the scale demonstrated good reliability and validity among Chinese teenagers (54). In the present study, the Cronbach's alpha for this scale was 0.75 at Time 1.

### Traditional bullying at Time 2

The Olweus Bully Questionnaire was used to evaluate the frequency of traditional bullying in the past six months (94). The questionnaire has six items, which consist of three dimensions: physical bullying (e.g., “We deliberately beat, kicked, pushed, bumped, or threatened a classmate”), verbal bullying (e.g., “We gave others an ugly nickname, scolded, teased, or satirized each other”), and relationship bullying (e.g., “We spread some rumors about a classmate and tried to make others dislike him or her”). There are two items under each dimension on a 5-point Likert scale from 1 (never happened) to 5 (several times a week). Higher average scores predicted higher levels of bullying among teens. This questionnaire has already exhibited good reliability in a previous study (95). Its Cronbach's alpha for this study was 0.78 at Time 2.

### Internet gaming disorder at Time 2

Adolescent IGD symptoms were measured with the eleven-item Internet Gaming Disorder Questionnaire (47, 96). All of the participants were required to respond to these items on a 3-point Likert scale ranging from 0 (never) to 2 (often) (e.g., “Have you played video games to avoid problems or bad feelings?” and “Have you ever spent too much time on video games to get poor grades or perform poorly in exams?”). All responses were recoded as 0 = “never,” 0.5 = “sometimes,” and 1 = “often.” Higher average scores reflect the greater possibility of IGD. Because this method of scoring takes into account participants who “occasionally” experienced symptoms, it is more accurate (47). The questionnaire has shown good reliability and validity in Chinese adolescents (42). The Cronbach's alpha for this questionnaire was 0.87 at Time 2.

### Covariates at Time 1

Demographic variables were collected as covariates in this study, including age, sex (1 = boys, 2 = girls), grade (1 = junior high school in grade 1, 2 = junior high school in grade 2, 3 = junior high school in grade 3, 4 = senior high school in grade 1, 5 = senior high school in grade 2, 6 = senior high school in grade 3), family economic status (1 = under the average level, 2 = equal to the average level, 3 = above the average level), and only child (1 = yes, 2 = no). The significant relationship between these demographic variables and the main study variables has been confirmed in earlier studies (49, 50, 97, 98).

### Data analyses

SPSS 25.0 and MPLUS 8.3 were used for data analysis. Initially, this study conducted preliminary analyses, including attrition analysis and common method bias analyses. Second, descriptive statistics and correlations of the key study variables and covariates were calculated with SPSS 25.0. Third, the hypothesized model was tested using structural equation modeling (SEM) with latent variables via MPLUS 8.3 (99). According to Wen and Ye (100), we evaluated the measurement model using confirmatory factor analysis (CFA) at the first step, and we constructed the SEM from the independent variable (T1 school assets) to the dependent variables (T2 traditional bullying and T2 IGD) based on controlling for covariates at the second step, then further constructed the SEM after adding the mediating variable (T1 self-control). The indices chosen for the study to evaluate good model fit include: χ^2^*/df* < 5, *CFI* and *TLI* >0.9, and *RMSEA* and *SRMR* < 0.08 (101, 102). Moreover, the bias-corrected percentile bootstrap method could accurately estimate the Type I error rates and test the significance of the mediation effect (103), and we examined the indirect effects using the standard errors and confidence intervals (CIs) with 5,000 bootstrap resamples. If both the upper and lower boundaries of the 95% confidence intervals (CIs) did not contain 0, it indicated that the result was statistically significant (104). Fourth, we employed the latent moderated structural (LMS) equation to test the moderating effect of T1 ISR (105). The advantages of the LMS are that it does not require artificially constructed product indicators and the interaction obeys normal distribution, thus effectively avoiding inconsistent parameter estimates due to different product indicator generation strategies and estimation errors resulting from non-normal interaction (106). Considering that this method does not provide the traditional fit indices mentioned above, this study examined the moderated mediation model fit by comparing the *AIC* and the likelihood ratio test of the baseline model without the latent moderating variable to the model with the latent moderating variable (107). Finally, this study used simple slope analysis to check the interaction effect (108).

## Results

### Preliminary analyses

Firstly, the independent sample *t*-test and chi-square test were used to compare the differences in covariates and key study variables among all the students who had provided data for two measurements and those who dropped out at Time 2. Independent sample *t*-test results exhibited that there was no significant difference between the two groups of participants in T1 school assets (*t* = −0.13, *p* = 0.90), T1 ISR (*t* = −1.86, *p* = 0.06), and T1 self-control (*t* = −0.48, *p* = 0.63). In addition, Chi-square test results showed that these two groups did not differ in family economic status (χ^2^ = 2.46, *p* = 0.29) and only child (χ^2^ = 0.14, *p* = 0.71), stating that the dataset of our research would not be affected because of attrition.

Secondly, since the data is all from subjective reports, there may be a problem of common method bias. According to previous studies, our study controlled the potential common method bias in the process of measurement by reversing the scoring of some items. Then, we tested the data for common method bias effects using Harman's single factor test (109). The results showed that there were 10 factors with eigenvalues greater than one, and the largest factor accounted for 20.64% of the total variance, less than the critical standard value of 40.00%. Therefore, it demonstrates that there is no serious influence on the data of this study due to common method biases.

The results of the descriptive statistics and correlations are exhibited in Table 1. The results indicated that there were significant correlations between T1 school assets, T1 ISR, T1 self-control, T2 traditional bullying, and T2 IGD (*ps* < 0.001). Specifically, positive associations were reported among T1 school assets, T1 ISR, and T1 self-control. T2 traditional bullying and T2 IGD were negatively correlated with T1 school assets, T1 ISR, and T1 self-control. And T2 traditional bullying was positively associated with T2 IGD. Moreover, age, sex, grade, family economic status, and only child were found to be significantly related to some of the key study variables (*ps* < 0.05). Thus, these variables were all included as covariates when testing the hypothesized model in subsequent steps.

**Table 1:** Descriptive statistics and correlations among variables.

**Study variables**	***M***	***SD***	***Skewness***	***Kurtosis***	**1**	**2**	**3**	**4**	**5**
1. T1 SA	3.40	0.49	−0.75	0.07	1				
2. T1 ISR	3.81	0.62	−0.11	−0.09	0.54^***^	1			
3. T1 SC	3.27	0.56	0.09	0.26	0.46^***^	0.43^***^	75		
4. T2 TB	1.11	0.30	4.26	21.89	−00.17^***^	−0.13^***^	−0.20^***^	1	
5. T2 IGD	0.11	0.15	2.14	5.85	−00.26^***^	−0.19^***^	−0.31^***^	0.34^***^	1
**Covariates**									
6. T1 Age	13.88	1.99	0.66	−1.19	−00.29^***^	−0.15^***^	−0.30^***^	0.09^*^	0.11^**^
7. T1 Sex	53.20^a^	–	–	–	0.08^*^	0.02	−0.01	−0.15^***^	−0.21^***^
8. T1 Grade	70.20^b^	–	–	–	−00.30^***^	−0.14^***^	−0.31^***^	0.09^*^	0.10^**^
9. T1 FES	92.50^c^	–	–	–	0.11^**^	0.09^*^	0.07^*^	−0.07^*^	0.05
10. T1 OC	50.90^d^	–	–	–	−00.06	−0.08^*^	−0.07^*^	−0.02	0.01

*N* = 742. SA, School Assets; ISR, Intentional Self-Regulation; SC, Self-Control; TB, Traditional Bullying; IGD, Internet Gaming Disorder.

a The percentage of boys;

b The percentage of participants who are junior high school students;

c The percentage of family economic status equal to the average level;

d The percentage of participants who are only children.

* *p* < 0.05,

** *p* < 0.01,

*** *p* < 0.001.

### Testing for mediation effect

Before testing the mediation model, we used CFA to explore the measurement model. The measurement model was composed of five latent variables, including T1 school assets, T1 ISR, T1 self-control, T2 traditional bullying, and T2 IGD. Among them, the measures of T1 ISR and T2 traditional bullying are both multidimensional. T1 ISR includes the three observed variables of goal selection, goal optimization, and goal compensation. T2 traditional bullying also includes the three observed variables of physical bullying, verbal bullying, and relationship bullying. Given that the scales of T1 school assets, T1 self-control, and T2 IGD were single-dimensional, we adopted the item-structure balance method to divide the items for all three scales into three observed variables, respectively (110). This method was effective in reducing intra-group differences, increasing metric consistency, and ultimately improving the model fit (111). The result showed that the measurement model fit well: χ^2^/*df* = 2.64, *CFI* = 0.98, *TLI* = 0.97, *RMSEA* = 0.05, and *SRMR* = 0.03.

**Figure 3:**
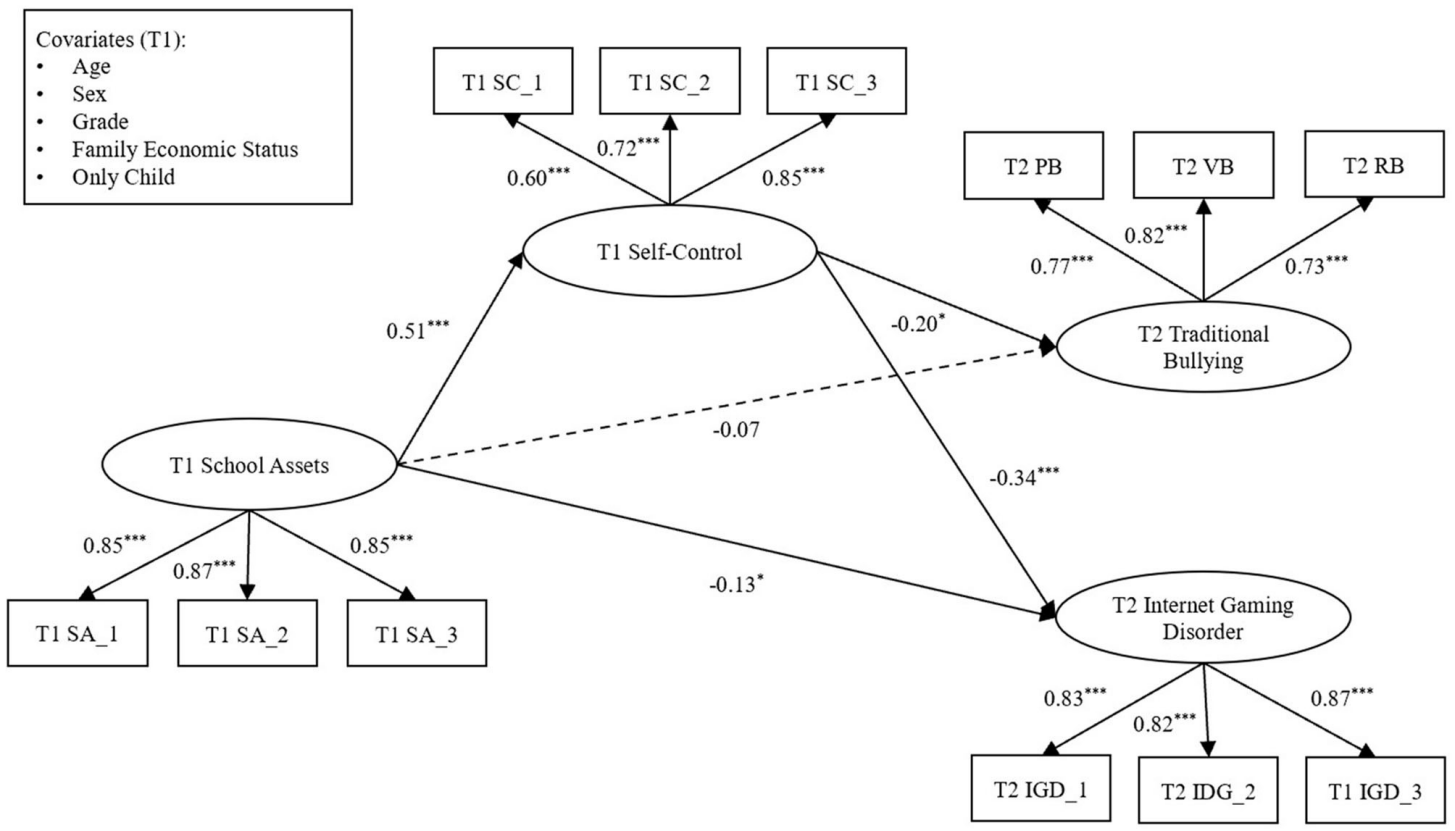
The results of mediation analysis. All estimated parameters are standardized. Covariates were omitted in the presentation. ^*^p < 0.05, ^***^p < 0.001.

Based on incorporating the covariates, this study conducted the mediation effect analyses. First of all, the result of the direct effect from the independent variable to the dependent variables showed that χ^2^/*df* = 3.72, *CFI* = 0.96, *TLI* = 0.94, *RMSEA* = 0.06, and *SRMR* = 0.08, indicating that the model fit the data well. T1 school assets played a negative role in T2 traditional bullying (β = −0.17, *p* < 0.001) and T2 IGD (β = −0.31, *p* < 0.001). Second, we added the mediator to the SEM. The results revealed that χ^2^/*df* = 2.86, *CFI* = 0.96, *TLI* = 0.95, *RMSEA* = 0.05, and *SRMR* = 0.07, indicating a good fit of the model. As presented in Figure 3, T1 school assets positively predicted T1 self-control (β = 0.51, *p* < 0.001). T1 self-control was a negative predictor of T2 traditional bullying (β = −0.20, *p* = 0.01) and T2 IGD (β = −0.34, *p* < 0.001). Finally, T1 school assets had a significant effect on T2 IGD (β = −0.13, *p* = 0.02) but no significant effect on T2 traditional bullying (β = −0.07, *p* = 0.28). As shown in Table 2, the values and the 95% confidence intervals (CIs) of the indirect effect are reported. The mediating role of T1 self-control between T1 school assets and T2 traditional bullying was significant (*B* = −0.04, *S.E*. = 0.02, 95%CI [−0.08, −0.01]). Then, T1 school assets also indirectly affected T2 IGD through T1 self-control (*B* = −0.06, *S.E*. = 0.01, 95%CI [−0.08, −0.04]).

**Table 2:** Indirect effects based on bias-corrected bootstrap estimates.

**Effects**	***Estimate***	***S.E*.**	***Est./S.E*.**	***Lower 2.5%***	***Upper 2.5%***
Total	−0.17	0.03	−5.68^***^	−0.24	−0.12
Total indirect	−0.10	0.02	−4.30^***^	−0.15	−0.06
Ind1: T1SA → T1SC → T2TB	−0.04	0.02	−2.49^*^	−0.08	−0.01
Ind2: T1SA → T1SC → T2IGD	−0.06	0.01	−4.98^***^	−0.08	−0.04

*N* = 742. SA, School Assets; SC, Self-Control; TB, Traditional Bullying; IGD, Internet Gaming Disorder. Bootstrap sample size = 5000.

* *p* < 0.05,

*** *p* < 0.001.

### Testing for moderated mediation effect

This study further analyzed the latent moderating effect of T1 ISR on the above mediation model using the LMS (105). We tested the baseline model without the latent moderating variable. The result revealed that χ^2^/*df* = 2.80, *CFI* = 0.95, *TLI* = 0.94, *RMSEA* = 0.05, and *SRMR* = 0.07, showing a good fit of the data. Then, we tested the moderated mediation model with the latent moderating variable. According to Fang and Wen (107), the results of the LMS did not provide fit indices such as *CFI* and *TLI*, so we examined whether the models fit the data well by comparing the *AIC* and likelihood ratio test of the two models. First, compared to the baseline model (*AIC* = 26,323.12), the *AIC* of the moderated mediation model (*AIC* = 26,307.18) was reduced by 15.94, showing that the fit indices of the latter were improved. Second, we conducted the likelihood ratio test of the baseline model (log likelihood = −13,094.56, *df* = 67) and the moderated mediation model (log likelihood = −13,085.59, *df* = 68). The result of the likelihood ratio test was significant (the log likelihood difference value of *D* = 17.94, Δ*df* = 1, *p* < 0.001), suggesting that the moderated mediation model was better than the baseline model. In summary, the moderated mediation model was acceptable.

**Figure 4:**
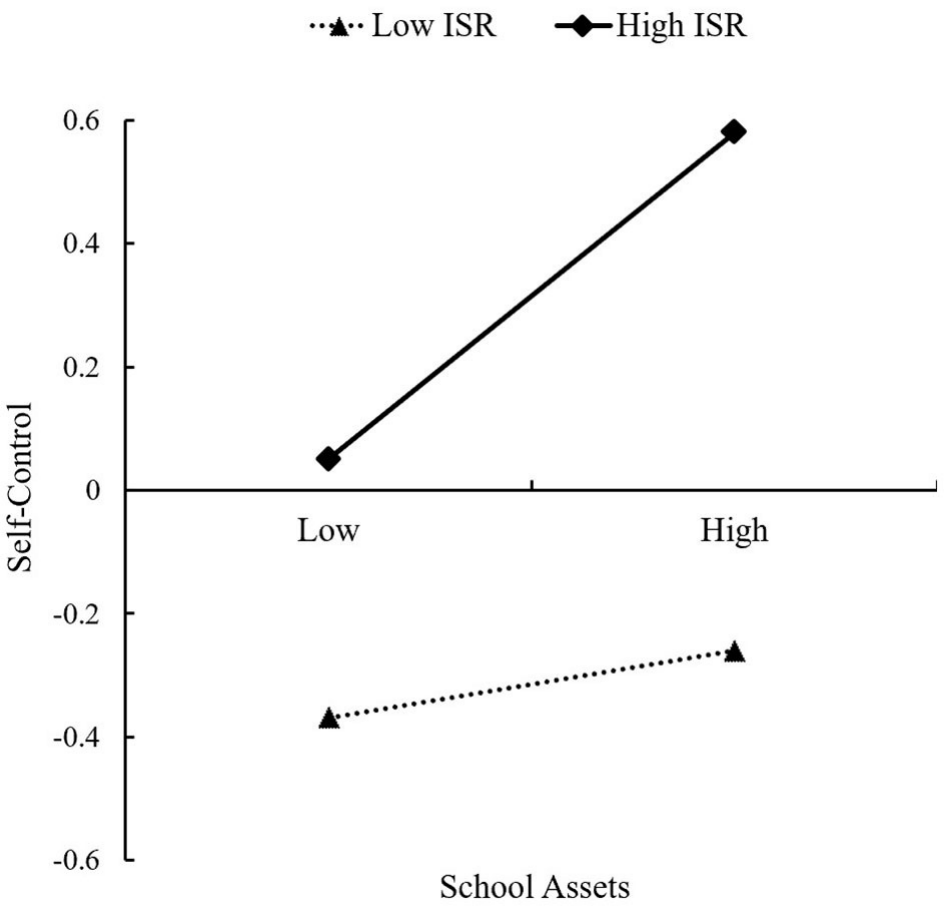
Interaction effects of school assets × ISR on self-control at Time 1.

The results revealed that the interaction effect of T1 school assets and T1 ISR had a significant effect on T1 self-control (*B* = 0.11, *p* < 0.001) (see Table 3). These suggested that T1 ISR positively moderated the predictive effect of T1 school assets on T1 self-control, as well as the two mediating paths of “T1 school assets → T1 self-control → T2 traditional bullying” (ind1) and “T1 school assets → T1 self-control → T2 IGD” (ind2). For indirect paths, when T1 ISR was 1 SD below the mean (i.e., low T1 ISR), the mediating effects were not significant (ind1: *B* = −0.01, *S.E*. = 0.01, 95%CI [−0.05, 0.003]; ind2: *B* = −0.03, *S.E*. = 0.02, 95%CI [−0.07, 0.10]); and when T1 ISR was 1 SD above the mean (i.e., high T1 ISR), the mediating effects were significant (ind1: *B* = −0.07, *S.E*. = 0.03, 95%CI [−0.15, −0.03]; ind2: *B* = −0.12, *S.E*. = 0.03, 95%CI [−0.21, −0.07]). Furthermore, in order to better understand the specific pattern of moderating effect, we used simple slope analysis to explore the interaction effect. As displayed in Figure 4, the effect of T1 school assets on T1 self-control was nonsignificant for adolescents with low T1 ISR (*B* = 0.05, *S.E*. = 0.04, *p* = 0.18), but significant for adolescents with high T1 ISR (*B* = 0.27, *S.E*. = 0.06, *p* < 0.001). Therefore, T1 ISR plays a promoting role, and the positive effect of T1 school assets on T1 self-control is increased with higher T1 ISR. Namely, the interaction pattern is consistent with the view of the predictive-enhancing hypothesis.

**Table 3:** Moderated mediation model results with T1ISR as a moderator.

**Outcome**	**Predictors**	***Estimate***	***S.E*.**	***Est./S.E*.**	***Lower 2.5%***	***Upper 2.5%***
T1 SC	T1 SA	0.16	0.04	3.80^***^	0.08	0.25
	T1 ISR	0.31	0.04	7.64^***^	0.24	0.40
	T1 SA^*^ISR	0.11	0.03	4.05^***^	0.06	0.16
**Effects**	ISR
Ind1: T1SA → T1SC → T2TB	M-1SD	−0.01	0.01	−1.19	−0.05	0.003
	M	−0.04	0.02	−2.29^*^	−0.10	−0.02
	M+1SD	−0.07	0.03	−2.44^*^	−0.15	−0.03
Ind2: T1SA → T1SC → T2IGD	M-1SD	−0.03	0.02	−1.26	−0.07	0.10
	M	−0.07	0.02	−3.16^**^	−0.13	−0.04
	M+1SD	−0.12	0.03	−3.73^***^	−0.21	−0.07

*N* = 742. SA, School Assets; ISR, Intentional Self-Regulation; SC, Self-Control; TB, Traditional Bullying; IGD, Internet Gaming Disorder. Bootstrap sample size = 5000. ^*^p < 0.05, ^**^p < 0.01, ^***^p < 0.001.

## Discussion

This study constructs the moderated mediation model by employing a two-wave study design to clarify not only how school assets influence adolescent traditional bullying and IGD (the mediating role of self-control), but also to provide a response to the question of under what conditions school assets and internal mechanisms of action have a more significant impact on traditional bullying and IGD (the moderating role of ISR). The results found that adolescents with superior school assets could reduce traditional bullying and IGD directly or indirectly through increased self-control, particularly among individuals with higher ISR. The hypothesized model of this study was confirmed, and the following sections discuss these findings in detail.

### School assets, traditional bullying, and internet gaming disorder

The results indicated that school assets negatively predicted adolescent traditional bullying and IGD, confirming hypothesis 1. This implied that premium school assets could effectively diminish the risk of traditional bullying and IGD among adolescents. As a result, adolescents with more assets are less likely to participate in hazardous behavior and more likely to engage in constructive behavior, which supports the stacking effect assumption of the developmental assets framework (34). As expected, school assets have been proven to be a strong protective factor against adolescent traditional bullying and IGD. Furthermore, according to stage-environment fit theory (112), adolescents may experience negative developmental outcomes if the assets afforded them in the school context do not fit their intrinsic needs to mitigate negative emotions or successfully cope with setbacks. Then, the likelihood of them improving their feelings of control and peer status by bullying others and coping with frustration and escaping reality by playing online games may increase (113, 114), ultimately leading to bullying and IGD. For this reason, giving young people as much experience and access to school assets as possible can be an essential part of preventing traditional bullying and IGD. The more school assets young people have, the fewer developmental problems they will encounter and the healthier they will grow up. This suggests that educators need to build a comprehensive system of school assets for students, which can be extremely helpful in preventing and decreasing traditional bullying and IGD.

### The mediating role of self-control

The results of this study demonstrated that self-control played a significant mediating role in the association between school assets and traditional bullying and between school assets and IGD as well, verifying hypotheses 2 and 3. Well-developed assets could indirectly affect negative developmental outcomes through self-control, consistent with previous studies (49, 56). In terms of the first half of the path of the mediating effect, school assets positively predicted self-control. The finding supports the strength model of self-control (51), which indicates that students with adequate school assets do develop better self-control, as external assets compensate to some extent for the resources that adolescents lose in controlling themselves. This reflected the crucial contribution of the school context to the development of individual self-control (55). In the second half of the mediating effect, self-control helped keep teenagers away from bullying and IGD. These results are in accordance with the viewpoint of Gottfredson and Hirschi (59), supporting the universality of the general theory of crime for explaining problem behaviors. First, self-control negatively predicted traditional bullying, similar to previous studies (24, 65). Namely, adolescents with lower self-control are more likely to exhibit behaviors associated with bullying others. Moreover, in a study of an exercise intervention to reduce aggressive behavior, improving adolescent self-control was a pivotal prerequisite for achieving the intervention effect (63). Second, higher self-control tended to predict lower IGD, which agrees with previous research (75, 76). Dong and Potenza (69) also found that the enhancement of individuals' own cognitive control may contribute to reducing the risk of IGD. It followed that individuals with higher self-control were often more capable of accomplishing behaviors that altered or dampened activities (115). Consequently, it is important to provide a wide range of school assets for youth and help them improve self-control to buffer traditional bullying and IGD. This suggests that practitioners should use home-school interventions as the foundation to focus on the interventions that can effectively strengthen self-control, such as exercise training and mindfulness therapies (116). On the other hand, teachers and parents should foster self-control abilities and offer specific strategies for adolescents to promote self-control.

### The moderating role of intentional self-regulation

In the current study, the moderating role of ISR in the effect of school assets on self-control was found to be significant, conforming to hypotheses 4 and 5. Notably, previous studies have already investigated the moderating role of ISR between positive assets (e.g., school climate) and problem behaviors and found that ISR acts as a buffer (44, 81). However, in distinction to the above studies, the present study intentionally extended the scope of the moderating role of ISR to positive developmental consequences, and the results confirmed that ISR is indeed also an appropriate moderating mechanism for enhancing adolescent self-control, which is more in line with the PYD perspective that emphasizes “cultivation and excavation” rather than “treatment and intervention”. The moderating effect verifies the predictive-enhancing model (87, 88). This viewpoint holds that increased school assets most benefit individuals with high levels of ISR; conversely, increased ISR most benefits individuals with high levels of school assets (86). In other words, given the same level of school assets, adolescents with higher ISR develop more self-control. Meanwhile, with increased self-control, ISR further magnifies the protective effects of school assets on both traditional bullying and IGD. This result also confirms the view of developmental systems theory (78), suggesting a simultaneous focus on creating assets and environments suitable for youth development. In this manner, internal (e.g., ISR) and external (e.g., school assets) assets are integrated to maximize the enhancement of constructive abilities and minimize unhealthy behaviors, finally enabling them to achieve positive development. Furthermore, Stefánsson et al. (117) have reported that ISR interacts with positive school assets and that ISR skills are an important factor in promoting school engagement. Therefore, in addition to offering excellent school assets, educators should also design training programs to advance the development of ISR skills in teenagers.

### Implications and limitations

This study has important theoretical implications. Our study provides a new and broader perspective for a more thorough understanding of adolescent traditional bullying and IGD. It embodies a shift from “problem intervention” to “exploring strengths” and deepens research in the area of adolescent traditional bullying and IGD. Furthermore, this study has profound practical implications. First, this study argues for asset building as a strategy for positive youth development, namely, to build capacity and increase wellbeing by enhancing school assets for youth. Secondly, researchers should focus on an “unlocking potential” approach to viewing adolescent problem behaviors. We found that school assets were effective in alleviating traditional bullying and IGD. Thus, schools can help adolescents avoid problem behaviors by maintaining a safe, positive, and healthy school environment. Practical activities and quality expansions can also be used to strengthen home-school links and improve students' school connectedness. In addition, teachers need to communicate with students on an equal footing and directly, which will help increase their sense of initiative and self-efficacy. Thirdly, we found a positive correlation between traditional bullying and IGD. This suggests that teachers and parents should not only pay more attention to the phenomenon of bullying and other negative peer relationships in schools and provide timely guidance to adolescents who engage in bullying, but also conduct more psycho-educational sessions on electronic media or internet use to help adolescents recognize the harm of IGD. Fourth, both self-control and ISR are internal assets that help adolescents cope with various aspects of developmental challenges. Parents and teachers should consciously use appropriate methods to cultivate and develop these skills. And schools need to develop mental health education courses for the whole school population to provide scientific knowledge and methods to promote self-control and ISR skills.

Despite the abundant findings of this study, there are still some limitations. Firstly, the data in our study was derived from subjective self-reports, which may have certain biases such as social expectations and personal motivations. Future studies could collect data through more channels, such as participants' parents, teachers, and peers, to increase the objectivity of the data. Secondly, this study used only a two-wave study design, which was tested less frequently and may have contributed to biased results. In the future, longitudinal studies lasting three or more waves should be used to better explore the causal relationships between variables. Thirdly, all variables in this study were not measured at two time points, which may have led to the loss of some important information necessary to fully grasp the association between variables. Future studies should measure the main study variables comprehensively at every time point and explore them deeply, such as using cross-lagged panel analysis to construct a longitudinal mediation model, taking into account both autoregressive and cross-lagged effects. Fourth, our data is only from Hubei Province. So, the findings of our study cannot accurately represent the development of the youth population nationwide. Future research should focus on the collection of national and cross-cultural data in order to obtain more general findings. Finally, this study only considered the role of the school system in traditional bullying and IGD. Experiencing cumulative assets in multiple ecological contexts tends to be more highly correlated with positive developmental outcomes than having assets in a single context (118). In future research, the influence of cumulative assets on adolescent traditional bullying and IGD could be investigated. It would be beneficial to test the asset-building approach to positive adolescent development from a more integrated perspective.

## Data availability statement

The raw data supporting the conclusions of this article will be made available by the authors, without undue reservation.

## Ethics statement

The studies involving human participants were reviewed and approved by the Research Ethics Committee of the College of Education and Sports Sciences, Yangtze University. Written informed consent to participate in this study was provided by the participants' legal guardian/next of kin.

## Author contributions

K-NQ collected and analyzed the data and drafted and revised the manuscript. XG contributed to the conception and design of this study. All authors contributed to the article and approved the submitted version.
